# “Defect-in-the-defect sign” after non-exposed endoscopic full-thickness resection

**DOI:** 10.1055/a-2598-4972

**Published:** 2025-06-03

**Authors:** Chung-Ying Lee, Kuang-I Fu, Ding-Ek Toh, Ming-Yao Chen, Yen-Ying Chen

**Affiliations:** 1 Division of Gastroenterology and Hepatology, Department of Internal Medicine, Shuang Ho Hospital, Taipei Medical University, New Taipei City, Taiwan; 2 Division of Gastroenterology and Hepatology, Department of Internal Medicine, School of Medicine, College of Medicine, Taipei Medical University, Taipei, Taiwan; 3 TMU Research Center for Digestive Medicine, Taipei Medical University, Taipei, Taiwan; 4 692411 Department of Endoscopy, Kamma Memorial Hospital, Tochigi, Japan; 5 14351 Department of Gastroenterology, Flinders Medical Centre, Adelaide, Australia; 6 499996 Department of Pathology, Shuang Ho Hospital, Taipei Medical University, New Taipei City, Taiwan; 7 Department of Pathology, School of Medicine, College of Medicine, Taipei Medical University, Taipei, Taiwan

Non-exposed endoscopic full-thickness resection (EFTR) has become a valuable technique for managing colorectal cancers (CRCs) with suspected deep invasion, particularly when conventional endoscopic resection is insufficient for safely obtaining adequate tissue beyond the submucosal layer. It enables en bloc resection while preserving GI wall integrity, with studies reporting high R0 resection rates and diagnostic accuracy, aiding treatment decisions for T1 CRCs, especially for those 20 mm or smaller 1 2 3 4 . Still, CRCs harboring far deeper invasion beyond endoscopic expectations could exist. Here, we present a unique case that revealed unexpectedly deep invasion (T4a) after non-exposed EFTR using a Padlock Clip.

During surveillance colonoscopy, a 75-year-old man was incidentally found to have a 12-mm depressed transverse colon lesion (IIa+IIc) ( Fig. 1 ). Image-enhanced endoscopy with magnification showed JNET classification type 3 within the depressed area, suggesting deep submucosal invasion (T1b). Abdominal CT revealed no lymph node involvement or distant metastasis. After shared decision-making, EFTR using a Padlock Clip (Aponos Medical) was performed ( Video 1 ).

**Fig. 1:**
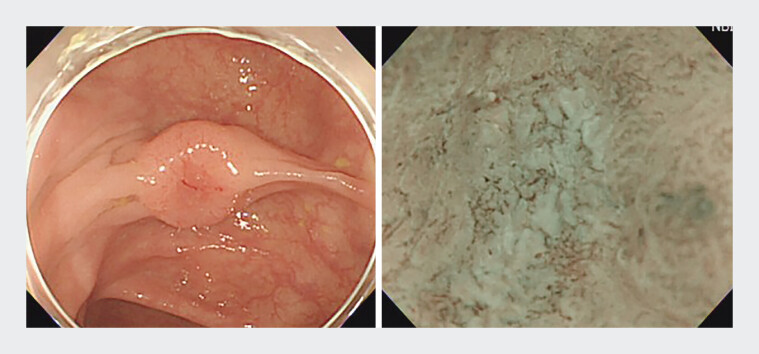
A 12 mm lesion (IIa+IIc, JNET classification type 3) with multiple-fold convergences was identified in the transverse colon.

“Defect-in-the-defect sign” after non-exposed EFTR for a colon lesion with deep invasion.Video 1

After resection, a deeper defect, “defect-in-the-defect sign”, was observed in the resected wound of exposed muscularis propria. Pathologically, the deeper defect corresponded to the site of unexpectedly advanced cancer extending beyond the serosal layer with lympho-vascular invasion ( Fig. 2 , left & right). The patient subsequently underwent laparoscopic left hemicolectomy 3 weeks after the endoscopic resection, and a 0.4 cm residual cancer was found in the pericolic tissue without lymph node metastasis, and thus it was finally determined to be stage II (T4aN0M0).

**Fig. 2:**
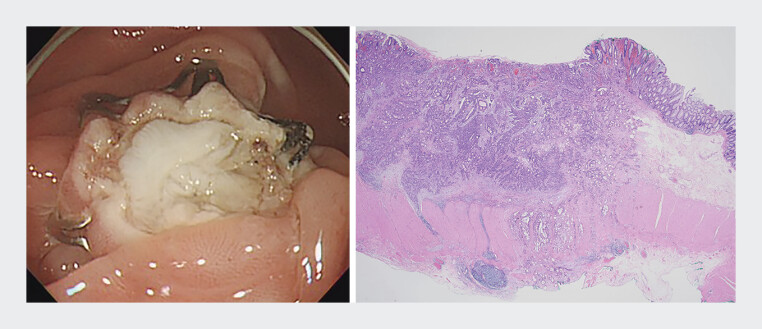
Endoscopic and histopathologic correlation of non-exposed EFTR. (Left) The post-resection defect demonstrates the “defect-in-the-defect sign.” (Right) Histopathologic analysis confirmed adenocarcinoma with visceral peritoneum invasion (pT4a), correlating with the unexpected deep invasion detected post-EFTR. Abbreviation: EFTR, endoscopic full-thickness resection.

As non-exposed EFTR gains broader clinical use, endoscopists should remain vigilant for cases where invasion depth exceeds expectations. This “defect-in-the-defect sign” could serve as a key endoscopic finding, indicating deeper cancer invasion beyond resection that warrants further detailed histological assessment to avoid underestimation or residual leading to fatal recurrence subsequently.

Endoscopy_UCTN_Code_TTT_1AQ_2AD_3AF

## Notes

Endoscopy E-Videos https://eref.thieme.de/e-videos
*E-Videos* is an open access online section of the journal *Endoscopy* , reporting on interesting cases and new techniques in gastroenterological endoscopy. All papers include a high-quality video and are published with a Creative Commons CC-BY license. Endoscopy E-Videos qualify for HINARI discounts and waivers and eligibility is automatically checked during the submission process. We grant 100% waivers to articles whose corresponding authors are based in Group A countries and 50% waivers to those who are based in Group B countries as classified by Research4Life (see: https://www.research4life.org/access/eligibility/ ). This section has its own submission website at https://mc.manuscriptcentral.com/e-videos .
